# Absence of anti-*Coxiella burnetii* antibodies in animal-hoarding individuals and their dogs living in a highly populated area

**DOI:** 10.1016/j.onehlt.2025.101169

**Published:** 2025-08-19

**Authors:** Danilo Alves França, Igor Silva Silito, Louise Bach Kmetiuk, Graziela Ribeiro da Cunha, Vivien Midori Morikawa, Helio Langoni, Marcelo Bahia Labruna, Alexander Welker Biondo

**Affiliations:** a Department of Preventive Veterinary Medicine and Animal Health, School of Veterinary Medicine and Animal Science, University of São Paulo, São Paulo, SP 05508-270, Brazil; b City Secretary of Health, Curitiba 80060-130, PR, Brazil; c Department of Veterinary Medicine, Federal University of Paraná State, Curitiba 80035-050, PR, Brazil; d City Secretary of Environment, Curitiba 80030-010, PR, Brazil; e Department of Animal Production and Preventive Veterinary Medicine, School of Veterinary Medicine and Animals Science, São Paulo State University, Botucatu 18618-681, SP, Brazil

**Keywords:** Q fever, Serology, Environmental exposure, Animal-hoarding, One health

## Abstract

This study aimed to investigate the presence of anti-*C. burnetii* antibodies in animal-hoarding owners and their dogs living in Curitiba, the eighth biggest city in Brazil with 1.8 million habitants. A total of 19 animal-hoarding individuals from 21 households and their 264 dogs were sampled. Serum samples were tested by the indirect immunofluorescence assay (IFA) for IgG antibodies against *C. burnetii*. Surprisingly, no hoarding owner or dog was seropositive, significantly contrasting (*p* = 0.0001) with the previous survey in policemen and their working dogs in the same city. The absence of seropositivity herein has indicated that, despite poor living conditions, hoarding dogs may not be exposed to *C. burnetii*, highlighting the role of environmental and behavioral isolation to the Q fever epidemiology. Thus, One Health approach to *C. burnetii* should always include concomitant human-animal serosurveys, essential to establish the pathogen cycle in different environments and contributing for effective control strategies.

## 1 Introduction

Q fever, caused by the *Coxiella burnetii* bacterium, has been considered a widely distributed zoonosis posting a significant challenge for global public health, particularly in areas with close human-animal interaction [1]. Studies have suggested that dogs can act as sentinels for the environmental presence of *C. burnetii* and potentially contribute to its transmission, particularly in densely populated urban settings and areas with inadequate waste management [2]. This dynamic is particularly relevant for stray dogs and shelter dogs, where poor hygiene conditions [2].

Animal hoarding has been classified as an obsessive-compulsive mental health disorder characterized by overwhelming accumulation and refusal to release animals and dispose of possessions, associated with increasing failure to provide adequate animal care [3]. The animal hoarding may be considered among the worst-case scenarios of “One unHealth”, due to increasing degradation of human, animal, and environmental health [3].

Despite the recent human-dog serosurveys of *C. burnetii* in vulnerable populations, no study to date has addressed *C. burnetii* infection in animal-hoarding owners and their dogs. Accordingly, this study aimed to assess the presence of anti-*C. burnetii* antibodies in animal-hoarding owners and their dogs.

## 2 Material and methods

The study herein was approved by the National Human Research Ethics Committee (protocol number 3.166.749/2019) and by the Animal Use Ethics Committee (protocol number 077/2015) of the Federal University of Paraná.

The city of study was Curitiba (25°25′47“S, 49°16′19” W), has been ranked nationwide as the 8th largest city, with a population of approximately 1.8 million habitants, the 6th in Gross Domestic Product (GDP), the 10th in Human Development Index (HDI) with 0.823 (high), and out of 5568 Brazilian cities.

At least 65 households with animal-hoarding cases were identified at the time in the urban area of Curitiba, totalizing 724 dogs. To determine the minimum sample required, a 50 % prevalence was considered, as disease prevalence in the country was unknown, a 95 % confidence level and a 5 % margin of error. The calculated result indicated at least 252 dogs of sampling.

All the participants were volunteers, signed a formal consent form and answered an epidemiological questionnaire before the samples were taken, which included important information for the epidemiology of Q fever.

Human and canine serum samples were tested using the indirect immunofluorescence assay (IFA), performed with Vero cells infected with *the C. burnetii* At12 strain antigen, originally isolated from *Amblyomma tigrinum* ticks in Argentina. This antigen has been validated and successfully applied in several studies conducted in Brazil, including clinical evaluations involving patients with confirmed acute Q fever [4,5], as well as serological investigations in various human populations and animal species [6]. Samples were tested at a screening dilution of 1:64, using fluorescein-conjugated antibodies (anti-human IgG and anti-dog IgG). Slides sensitized with the antigen received 10 μL of serum in each well and were incubated at 37 °C for 30 min in a humid chamber. After washing and drying, 10 μL of conjugated antibodies were applied to each well, followed by another 30-min incubation. To ensure the accuracy and consistency of the assay, each slide included positive and negative control sera, previously characterized during routine laboratory procedures [7]. All samples were processed, and fluorescence reading was performed using an Olympus BX53 immunofluorescence microscope with a 40× objective. Samples were considered positive when showing clear and homogeneous cytoplasmic fluorescence across the field.

Statistical analyses were performed using SAS Studio 3.81 (SAS Institute Inc., Cary, NC, USA) [8]. The significance level was set at *p* < 0.05.

## 3 Results

Owners of a total of 11 households agreed to take part in the study, and dog samples were collected from all of them. Residents' lack of interest and refuseness in taking part in the research lead to low human sampling, with only 11/65 (16.9 %) investigated households. Although the final participation involved 11 households, a total of 19 human individuals were sampled, as some households had more than one resident who voluntarily signed the consent form participation. Unfortunately, few owners authorized only the dog blood samplings, declining to provide their own blood samples. Thus, the final samplings included all available dogs from these 11 households and their owners who voluntary signed agreement to participate.

Epidemiological information such as the number of dogs in the house, the origin of the animals, the type of food provided, the water provided to the animals, the presence of ticks, the presence of another animal in the house and the accumulation of feces in the area were gathered and presented in Supplementary Material 1.

According to the serological results, no dog was seropositive for anti-*C. burnetii* antibodies. A Fisher's exact test showed a significant difference (*p* = 0.0001) between the prevalence in our study and the prevalence in the study of França et al. [7]. Similarly, no human participants tested positive.

## 4 Discussion

According to the study herein and others our research group have conducted in Brazil [6,9], dogs may not influence human transmission of Q fever or have been similarly exposed to infection sources, unlikely other reports worldwide [9,10].

Once serological testing has identified positive human and animal samples, molecular testing (PCR) on biological and environmental samples should be recommended to investigate active infection and potential exposure source. For instance, dog feces, vaginal swabs and household environmental samples (e.g., dust, soil) have been considered appropriate for detecting *C. burnetii* DNA, particularly in bitches with history of reproductive problems [2,11,12]. However, antibody absence herein indicated no prior pathogen exposure, substantially reducing the likelihood of active infection and bacterial shedding. Nevertheless, lack of biological samplings for molecular screening has been a limitation in the present study, and future surveys should consider such approach to better establish the Q fever transmission dynamics in animal-hoarding environments.

The absence of *C. burnetii* seropositivity in the dogs herein has contrasted significantly (*p* = 0.0001) with the prevalence found in police dogs sampled in the same city. This significant difference may be attributed to police dogs' daily activities, traveling, searching and other incursions. However, police dogs were neutered, unlike the dogs in this study, which may have prevented infection during mating encounters. In addition, police dogs had access into forests, agricultural and livestock areas, and dogs herein have mostly remained restricted indoors or into household yards, living in a full urban area and with no direct contact with livestock areas or of dense natural vegetation.

The fact that most dogs are not neutered and breed in households would be a risk factor for human and other animal infection. A human case of Q fever was directly linked to the infection of a parturient female dog, which resulted in pneumonia in three family members [12]. Studies of animal shelters around the world revealed positivity rates of 4.8 % (5/104) in Portugal and 1.9 % (5/265) in Australia [13,14]. Both contexts involve an accumulation of animals, with the distinction that shelters receive new street dogs more frequently and have stricter neutering protocols. It seems that the enclosed environment and lack of access to the street protects the animals from infection.

As a major limitation, tick and livestock (cattle, sheep, goats) contact as primary *C. burnetii* reservoirs in Q fever transmission was not investigated. Also, the epidemiological questionnaire was designed to zoonosis exposure in general instead of specifically Q fever. As a result, input data missed relevant variables to Q fever including livestock contact, birthing events, and dairy consumption of unpasteurized products. Since Q fever has been a significant reproductive disease, the lack of systematic collection of reproductive data in dogs (e.g., history of parturition, abortion, or contact with breeding animals) could be a critical bias if results were higher. Thus, future studies should always consider disease-specific questionnaires specifically directed to Q fever.

In addition, voluntary participation may have resulted in underrepresentation of certain populational groups (e.g., a higher likelihood of participation by owners of household dogs compared to stray/rural dogs). Random sampling methods (e.g., cluster or stratified sampling) should be strongly recommended for future research to ensure a more representative sample, despite difficulties on accessing hoarding populations.

The idea that exposure to *C. burnetii* goes far beyond contact with farm animals is reinforced by a recent study from the United Kingdom, which reported an outbreak of the disease in cardboard factory workers exposed to contaminated old wood materials [15]. Similarly, the incidence of Q fever in a prison in French Guiana demonstrates the complexity of this epidemiology, where environmental and human factors interact in a unique way [16]. These cases illustrate how Q fever can emerge in unexpected contexts, highlighting the importance of One Health approach to understand, prevent and mitigate transmission.

The absence of seropositivity observed herein may significantly contribute for the understanding of Q fever epidemiology, by indicating that dogs in hoarding environments, despite inadequate management conditions, may not play a relevant role in the *C. burnetii* transmission.

## CRediT authorship contribution statement

**Danilo Alves França:** Writing – review & editing, Writing – original draft, Methodology, Investigation, Formal analysis, Conceptualization. **Igor Silva Silito:** Writing – review & editing, Validation, Methodology, Formal analysis. **Louise Bach Kmetiuk:** Writing – review & editing, Writing – original draft, Visualization, Investigation. **Graziela Ribeiro da Cunha:** Writing – review & editing, Visualization, Methodology, Investigation, Data curation, Conceptualization. **Vivien Midori Morikawa:** Writing – review & editing, Data curation, Conceptualization. **Helio Langoni:** Writing – review & editing, Validation, Methodology, Investigation, Formal analysis. **Marcelo Bahia Labruna:** Writing – review & editing, Writing – original draft, Resources, Methodology, Investigation, Formal analysis, Conceptualization. **Alexander Welker Biondo:** Writing – review & editing, Writing – original draft, Visualization, Validation, Supervision, Resources, Project administration, Methodology, Investigation, Funding acquisition, Data curation, Conceptualization.

## Funding

No funding.

## Declaration of competing interest

The authors declare that they have no known competing financial interests or personal relationships that could have appeared to influence the work reported in this paper.

## Data Availability

Data will be made available on request.

## Appendix A Supplementary data

Supplementary Material Characteristics of the households and dogs participating in the study in Curitiba, Brazil, including the number of people and dogs per household, origin of the dogs, food provided, access to treated water, presence of ticks, other animals on the premises and feces management. Image 1
